# Feasibility of utilizing functional near-infrared spectroscopy to measure the cognitive load of paramedicine students undertaking high-acuity clinical simulations in Australia: a case study

**DOI:** 10.3352/jeehp.2024.21.38

**Published:** 2024-12-10

**Authors:** Jason Betson, Erich Christian Fein, David Long, Peter Horrocks

**Affiliations:** 1 Faculty of Health, Australian Catholic University, Melbourne, Australia; 2 School of Health and Medical Sciences and Centre for Health Research, University of Southern Queensland, Ipswich, Australia; 3 School of Psychology and Wellbeing, University of Southern Queensland, Toowoomba, Australia; 4 School of Clinical Sciences, Queensland University of Technology, Brisbane, Australia; Hallym University, Korea

**Keywords:** Cognition, Emergencies, High fidelity simulation training, Near-infrared spectroscopy, Paramedicine, Australia

## Abstract

**Purpose:**

Paramedicine education often uses high-fidelity simulations that mimic real-life emergencies. These experiences can trigger stress responses characterized by physiological changes, including alterations in cerebral blood flow and oxygenation. Functional near-infrared spectroscopy (fNIRS) is emerging as a promising tool for assessing cognitive stress in educational settings.

**Methods:**

Eight final-year undergraduate paramedicine students completed 2 high-acuity scenarios 7 days apart. Real-time continuous recording of cerebral blood flow and oxygenation levels in the prefrontal cortex was undertaken via fNIRS as a means of assessing neural activity during stressful scenarios.

**Results:**

fNIRS accurately determined periods of increased cerebral oxygenation when participants were undertaking highly technical skills or making significant clinical decisions.

**Conclusion:**

fNIRS holds potential for objectively measuring the cognitive load in undergraduate paramedicine students. By providing real-time insights into neurophysiological responses, fNIRS may enhance training outcomes in paramedicine programs and improve student well-being (Australian New Zealand Clinical Trials Registry: ACTRN12623001214628).

## Graphical abstract

[Fig f4-jeehp-21-38]

## Introduction

### Background

Paramedics, like emergency and intensive care nurses and doctors, are trained to assess and treat critically unwell patients. In healthcare settings, a high-acuity scenario refers to a situation where a patient’s condition is characterized by a high level of severity, complexity, and urgency [1]. Due to the often low exposure to high-acuity patients during clinical placement, high-fidelity simulations are frequently used as an andragogic tool by education providers. These scenarios generally involve patients who require intense monitoring, rapid interventions, and a high level of medical care due to the critical nature of their condition. This may lead to an elevated stress response, which can be overwhelming for the participant and lead to poor clinical performance [2].

Cognitive load can be defined as a multifactorial construct of mental workload imposed on a participant during a particular task [3,4]. Expanding further, the perceived pressure on working memory during task execution can be related back to the effort of thinking, learning, and reasoning—leading to a cognitive stress point [5]. To objectively determine cognitive load and therefore cognitive stress, a device measuring brain activity must be employed. Functional near-infrared spectroscopy (fNIRS) is a non-invasive neuroimaging technique that measures changes in blood oxygenation and blood volume in the brain [6]. The basic principle behind fNIRS involves the use of light to penetrate the skull and reach the brain tissue. Hemoglobin (Hb), the oxygen-carrying molecule in blood, absorbs light differently depending on its oxygenation state [7]. By measuring the changes in light absorption, fNIRS can provide information about the concentration of oxygenated and deoxygenated Hb (denoted by O_2_Hb and HHb, respectively) in the brain [8]. An increase in O_2_Hb concentration is associated with increased blood flow and oxygen delivery to active brain regions. Oxidative metabolism, the primary means of energy production in the brain, varies depending on hemodynamic changes (blood flow and volume) and brain activity [9]. The concentration of O_2_Hb can be interpreted as a marker of increased neural activity in those regions. This is consistent with active neurons requiring more oxygen to support their metabolic demands. An increase in the HHb concentration is typically associated with increased oxygen consumption in active brain regions; therefore, as neurons consume oxygen during activity, HHb levels rise. Interpreting fNIRS data involves analyzing changes in O_2_Hb and HHb levels in response to specific stimuli or tasks, providing an indirect and potentially valuable indicator of neural activity. To our knowledge, no research has been undertaken using fNIRS as a means of examining the neurological response during high-acuity clinical scenarios.

### Objectives

Our study aimed to assess fNIRS as a non-invasive neural monitoring modality for final-year undergraduate paramedicine students undertaking simulated high-acuity scenarios. We formed 2 hypotheses: (1) fNIRS data would display increased neural activity exhibited by increased concentrations of O_2_Hb during high-stress junctures of the clinical scenario; and (2) a repeat exposure to a comparable acuity scenario a short time after the first scenario would reduce neural activity and subsequently cognitive stress for participants, leading to improved clinical performance.

## Methods

### Ethics statement

Ethical approval was granted by the University of Southern Queensland’s Human Research Ethics Committee (HREC) on 26 August 2021 (reference number: H21REA158). Administrative review and ethics ratification were sought from the Australian Catholic University HREC and approved on 10 September 2021 (reference number: 2021-204R). This study forms a component of a larger trial registered on November 27, 2023 with the Australian New Zealand Clinical Trials Registry (ANZCTR) (allocation number: ACTRN12623001214628).

### Study design

This is a case study with a one-group post-test only design.

### Participants

Eight final-year undergraduate Bachelor of Paramedicine students volunteered for the study. To provide a consistent level of academic ability and required clinical knowledge, only final year students with a grade point average above 5.0 were considered for inclusion. Published exclusion criteria for participants included having been diagnosed anxiety or stress-related disorders, and taking medication that affects the central nervous system or cardiovascular system that could alter normal physiological responses to stress (e.g., medications for epilepsy, anxiety, mood disorders, sleeping tablets such as Stilnox, benzodiazepines, melatonin, or beta blockers). Applying these criteria excluded 3 potential volunteers. Participants registered their interest via an online anonymized research participation form and were contacted by phone to discuss the research project and their eligibility for the study assessed.

### Intervention

**Fig. 1.**
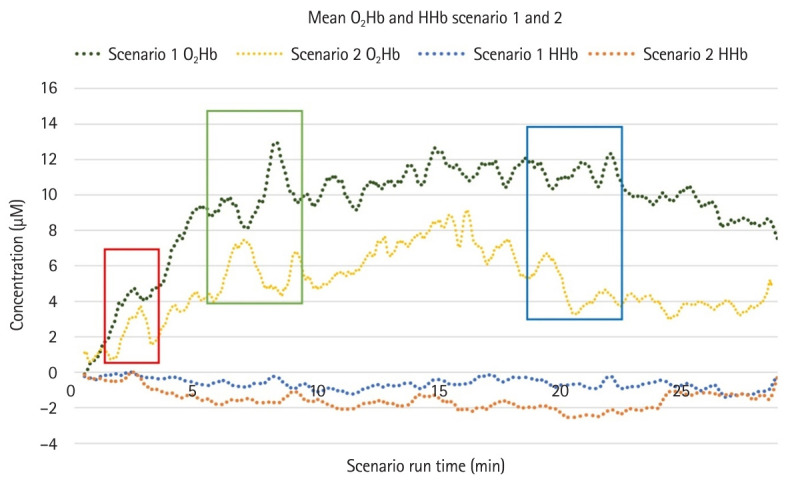
Mean O_2_Hb and HHb concentration (µM) levels across the duration of scenario 1 (n=8) and scenario 2 (n=8). Red box: early, green box: mid, blue box: late.

Two separate high-acuity scenarios were undertaken within the paramedicine practical laboratories of the university (Fig. 1). The mean atmospheric conditions within the practical laboratories were controlled to be as similar as possible across both scenarios and recorded as: temperature 23.3±0.5°C, relative humidity 35.2%±2.9%, noise level 46.9±1.9 dB, and illumination 463.6±172.2 lux.

**Fig. 2.**
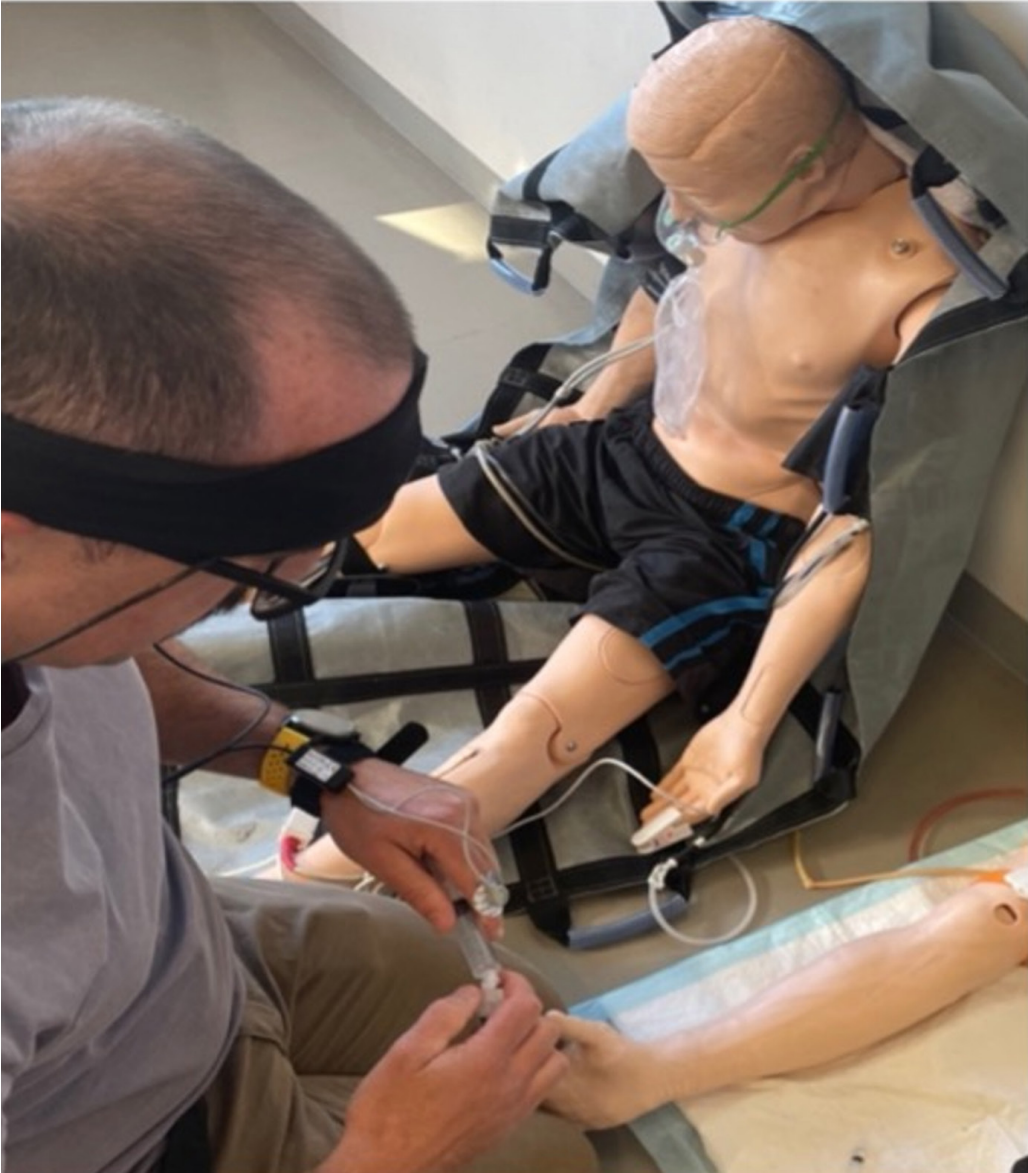
PortaLite MKII (Artinis Medical Systems, Netherlands) functional near-infrared spectroscopy system worn by a student undertaking a high-acuity scenario.

**Fig. 3.**
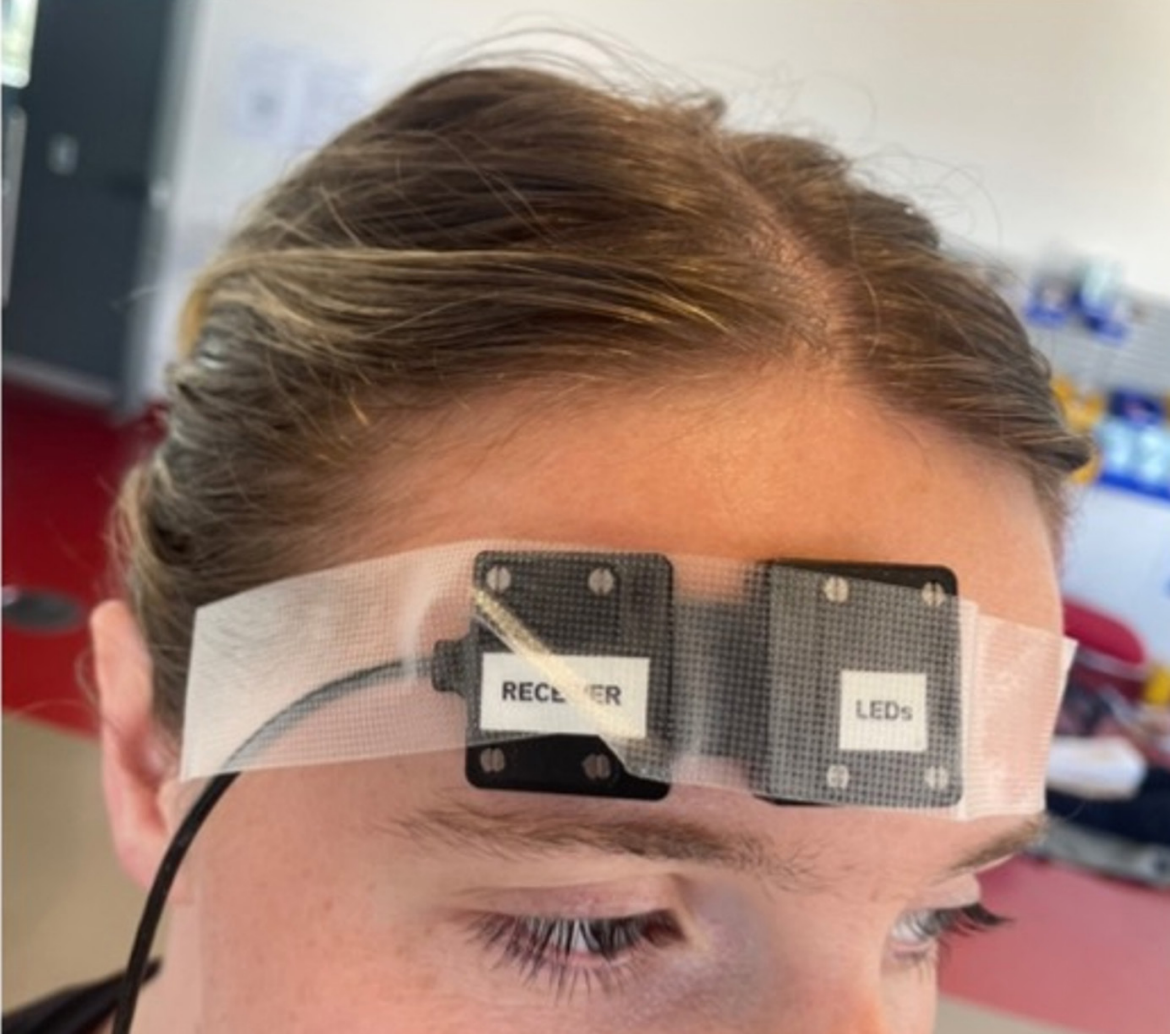
Sensors consisting of 3 light-emitting diodes were placed approximately 2 cm above the right eyebrow and continuously recorded data whilst students completed high-acuity scenarios (Informed consent was obtained from the student in the photo.).

High-acuity scenarios can create immediate stress and impose a large cognitive load as rapid clinical decisions are required. In our study, participants were informed prior to attending that the simulated patient would involve an adult medical patient, not in cardiac arrest and not suffering life-threatening trauma. Participants wore the PortaLite MKII (Artinis Medical Systems) fNIRS system (Figs. 2, 3).

A research team member, behind a one-way glass mirrored window, assessed each scenario using a standardized objective structured clinical exam (OSCE) grading template to allocate the participant a clinical performance score out of 10 (Supplement 1). The first scenario (scenario 1) involved assessment and management of a critically unwell anaphylaxis patient. The second scenario (scenario 2) centered around an unconscious patient secondary to a narcotic overdose. These scenarios were chosen as both require rapid clinical intervention shortly after scenario commencement for positive patient outcomes. Additionally, due to the nature of clinical placements, it is unlikely a student would have independently managed a similar case to establish pre-exposure, obviating familiarity with patient presentation as a confounding variable. All participants completed both scenarios solo at the same time of the day, 7 days apart with no advance knowledge of the patient presentation prior to the scenario commencement.

### Outcome variables

The mean O_2_Hb concentration across scenarios was the outcome variable.

### Data sources/measurement

#### fNIRS instrumentation and data processing

A 6-channel continuous wave PortaLite MKII (Artinis Medical Systems) fNIRS system utilizes the modified Beer-Lambert law with an age-dependent differential pathlength to map O_2_Hb, HHb, and total Hb concentration changes within cortical brain tissue. Sensors consisting of 3 light-emitting diodes, each with 2 wavelengths of 760 and 850 nm, and 2 photodiode receivers were placed approximately 2 cm above the right eyebrow as recommended by Artinis Medical Systems.

This location assesses blood flow to the prefrontal cortex via an inter-optode distance of 29, 35, and 41 mm with a sample rate if 100 Hz. Three channels each for O_2_Hb and HHb were acquired and processed via Oxysoft software ver. 3.2.72 (Artinis Medical Systems). A black sports headband covered the sensors to prevent ambient light interference, and the control unit and battery were placed in a belt around the waist. To obtain baseline, all participants were asked to sit perfectly still with their head straight and upright for 30 seconds prior to scenario commencement. Mean values were determined for the 3 channels of O_2_Hb and HHb and all data reported as variation from baseline.

Room atmospheric conditions were controlled to be as similar as possible. Temperature (°C), relative humidity (%), ambient noise (dB), and illumination (lux) were all recorded and monitored via a handheld 5-in-1 meter (model: LM-8102; Munro Instruments).

Research data are available at Dataset 1.

### Bias

No known selection biases were identified.

### Statistical method

Statistical analyses were conducted using IBM SPSS ver. 29.0 (IBM Corp.; running on Windows 10). All data were reported as means±standard deviation, and P<0.05 was deemed statistically significant. The paired-sample t-test with bootstrapping was undertaken for statistical analysis of data within a scenario, and these tests were applied to early, mid, and late time-points within the time series of data for each key variable.

## Results

A mean completion time of 30.8±2.1 minutes was recorded for both scenarios, with the minimum completion time of 25.5 minutes used for all calculations of the area under the curve presented in this study. The mean O_2_Hb concentration (µM) levels and standard deviation were calculated for scenarios 1 and 2, as shown in Table 1. No data were lost due to equipment error or participant withdrawal.

The mean HHb concentrations (µM) were also recorded, but did not vary significantly across the scenario duration. This agrees with literature demonstrating that in healthy people, minimal variations in HHb levels (comparative to O_2_Hb) are observed during episodes of acute cognitive stress [10]. When comparing O_2_Hb across both scenarios, we observed a significant decrease (P<0.001; 95% confidence interval [CI], 4.24-4.29) in mean O_2_Hb concentrations for scenario 2 compared to scenario 1 (Fig. 1). This was also reflected in a significant improvement in clinical scores from scenario 1 to scenario 2 (4.6±1.6 versus 7.5±1.1; P=0.02; 95% CI, –4.13 to –1.50).

Data obtained from the fNIRS device also identified peaks of neural blood flow around the 2–3-minute mark of each scenario and again around the 7–10-minute mark. These peaks, along with fluctuations around the 19–22-minute mark, have been determined as “early,” “mid,” and “late” scenario points indicated by colored boxes in Fig. 1. To determine if these variations were statistically significant, the paired-samples t-test was performed comparing data from these chosen points with full scenario data (Table 2).

## Discussion

### Interpretation

In our study relating to hypothesis 1, the early peak of O_2_Hb evident in Fig. 1 corresponded to the initial management of intramuscular adrenaline for anaphylaxis (scenario 1) and intramuscular naloxone for narcotic overdose (scenario 2). These peaks were significantly different from other time points in each scenario (scenario 1 “early” P=0.020 and scenario 2 “early” P=0.038). The data showed an identifiable increase in neural blood flow as participants undertook rapid patient assessment and history gathering concurrently with the technical skill of an intramuscular injection. The interpretation for final-year paramedicine students is that the early minutes of a high-acuity scenario present increased cognitive load as they multi-task several high-priority clinical tasks.

The second variation we observed, around the 7–9 minute mark, coincides with students undertaking a differential diagnosis and further advanced technical skills. In scenario 1, participants were obtaining intravenous access at this time point, with fNIRS demonstrating a large peak in neural blood flow. In scenario 2, 2 individual peaks were observed; a large early peak at around the 7-minute mark was influenced strongly by the 4 participants with an elevated stress response, and a more subtle second peak at approximately the 9 minute-mark was influenced by the other 4 participants. Reviewing the clinical assessment notes recorded at the time, the stressed group were at this time point considering other pharmacological interventions that were ultimately not required for the narcotic overdose. This resulted in a short period of high cognitive load during which participants were self-assessing whether they had correctly made appropriate clinical and pharmacological decisions. Conversely, in the less stressed group, a smaller and later fNIRS peak was observed around the 9–10-minute mark where participants were correctly reassessing the Glasgow Coma Scale of the simulated patient to decide on further ongoing treatment with naloxone. Examining the analysis of these designated time points (Table 2), another statistically significant variation was observed late in scenario 1 (P=0.007). Again, reviewing clinical notes from the scenario, it was at this point that participants were deciding on the administration of anti-emetic medication for the anaphylactic patient who had ingested an allergen orally. This juncture appeared to demand a larger amount of neural activity to form a clinical decision within the scenario. From the clinical outcome perspective of the scenario, administering or withholding the medication were both justifiable treatment pathways with no impact on the overall clinical score achieved. These results support hypothesis 1 and indicate that fNIRS technology is able to accurately identify periods of increased cognitive load during high-acuity clinical scenarios.

To assess hypothesis 2, we compared data for all 8 participants across both scenarios to see if a repeat exposure reduced cognitive stress and improved clinical performance (Table 1). Scenario 1 demonstrated a significantly larger fNIRS response than scenario 2, indicating that familiarity or comfortableness accompanied a repeated exposure to a comparable acuity scenario assessment. In approaching scenario two 7 days later, these participants who had been exposed to a similar high-acuity scenario, may have been somewhat more relaxed and therefore employed less cognitive load in their second attempt. We recognize that a small sample size renders any definitive results challenging to interpret, and indeed, some individual results may have skewed the data. When examining the clinical scores, fewer than 50% of participants passed scenario 1; however, scenario 2 saw a pass rate of 80%, which is a significant improvement in clinical performance. We again acknowledge that this is based on a small sample size of n=8, but this finding provides valuable insight for healthcare educators as to the benefit of repeated practice of simulated high-acuity scenarios.

Our results demonstrate that fNIRS can provide real-time data on cortical activation, making it suitable for capturing stress responses as they occur. Larger studies, incorporating other appropriate objective measures are recommended to validate fNIRS data relevant to the acute stress response.

### Limitations

Our small sample size of n=8 restricted any definitive statistically significant findings. However, with each participant completing 2 separate scenarios and the PortaLite MKII recording 6 channels (3 O_2_Hb and 3 HHb), we were able to gather 96 individual wavelengths of neural activity data. Each wavelength recorded a data point every 1/10th of second over an average scenario time of 30.8 minutes, meaning an exceptionally large pool of data was available for analysis. As a relatively new technology, some key limitations must also be considered. The spatial resolution of fNIRS is not as high as some other neuroimaging techniques, such as functional magnetic resonance imaging. This means that it may not provide precise information about the location of neural activity. Additionally, the near-infrared light used in fNIRS has limited penetration depth, mainly capturing signals from the cortical surface. This restricts the ability to study deeper brain structures. The signals measured by fNIRS are also influenced by scalp and skull tissue, with variability in individual anatomy potentially affecting the accuracy of measurements. The learning environment may also be a confounder to stress levels. If any participant had a previous failing OSCE result in the same laboratory space for which this experiment was undertaken, a priori experience may have caused elevated stress responses due to environmental triggers, potentially affecting the results.

### Conclusion

Approaching the completion of their studies, undergraduate paramedicine students have obtained significant amounts of clinical and technical knowledge. However, they may not have achieved the ability to recognize their own cognitive overload during high-acuity clinical presentations. This is also likely true for many other health disciplines, first responders and military personnel. By utilizing novel technologies such as fNIRS, educators can provide real-time non-invasive neural feedback to students as they practice simulation-based learning. This allows students to gain an appreciation of their own individual cognitive stress points and develop plans to mitigate overload. The addition of stress mitigation strategies to their clinical and technical knowledge will likely assist graduate paramedics to be better clinicians in real world medical situations.

## Figures and Tables

**Figure f4-jeehp-21-38:**
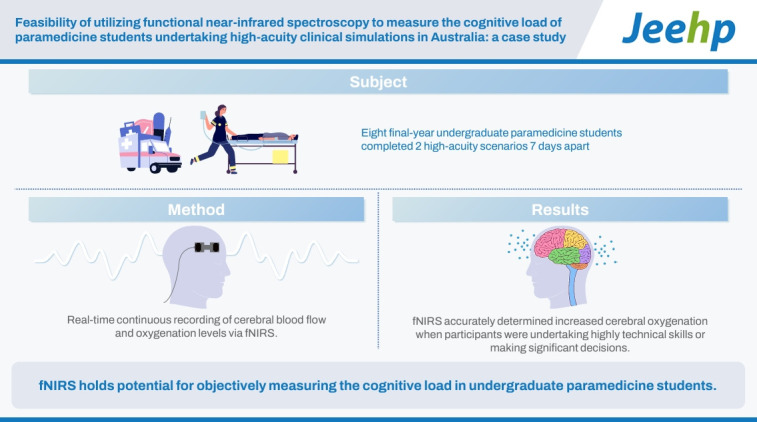


**Table 1.** O_2_Hb concentration (µM) levels and clinical performance

	Mean O_2_Hb concentration (µM)	Area under the curve	Clinical score/10
Scenario 1	9.3±4.9	232.03	4.6±1.6
Scenario 2	6.3±4.8	158.86	7.5±1.1

Values are presented as mean±standard deviation or number unless otherwise stated. Area under the curve: cumulative O_2_Hb concentration (µM). Scenario duration 25.5 minutes

**Table 2.** O_2_Hb concentrations (µM) at early, mid, and late junctures of each scenario

	Mean O_2_Hb concentration (µM)	Sig. (2-tailed) bootstrap (P-value)	t-value	95% Confidence interval
Scenario 1	9.3±4.9			6.357 to 12.656
Early scenario 1	4.4±2.9	0.020	–3.587	2.376 to 6.320
Mid scenario 1	10.3±6.0	0.222	1.304	6.152 to 13.982
Late scenario 1	11.3±6.0	0.007	3.943	7.777 to 15.541
Scenario 2	6.3±4.8			3.089 to 8.852
Early scenario 2	2.4±4.1	0.038	–3.044	–0.386 to 5.031
Mid scenario 2	5.7±4.4	0.758	–0.341	3.050 to 8.852
Late scenario 2	4.8±5.7	0.430	–0.885	1.053 to 8.561

Values are presented as mean±standard deviation or number unless otherwise stated. “Early,” “mid,” and “late” assessed against comparator of full scenario data for each scenario. P<0.05 was deemed statistically significant.

## Footnotes

**Data availability**
Data files are available from Harvard Dataverse: https://doi.org/10.7910/DVN/WL9HPY Dataset 1. Research data of this study.

## Supplementary materials

Supplementary files are available from Harvard Dataverse: https://doi.org/10.7910/DVN/WL9HPY

Supplement 1. Standardized objective structured clinical exam (OSCE) grading template.

Supplement 2. Audio recording of the abstract.

## References

[1] Chrimes N (2016). The Vortex: a universal ‘high-acuity implementation tool’ for emergency airway management. Br J Anaesth.

[2] Harvey A, Nathens AB, Bandiera G, Leblanc VR (2010). Threat and challenge: cognitive appraisal and stress responses in simulated trauma resuscitations. Med Educ.

[3] Paas FG, Van Merrienboer JJ (1993). The efficiency of instructional conditions: an approach to combine mental effort and performance measures. Hum Factors.

[4] Paas FG, Van Merrienboer JJ (1994). Instructional control of cognitive load in the training of complex cognitive tasks. Educ Psychol Rev.

[5] Haapalainen E, Kim S, Forlizzi JF, Dey AK (2010). Psycho-physiological measures for assessing cognitive load. https://doi.org/10.1145/1864349.1864395.

[6] Sevcenko N, Schopp B, Dresler T, Ehlis AC, Ninaus M, Moeller K, Gerjets P (2022). Neural correlates of cognitive load while playing an emergency simulation game: a functional near-infrared spectroscopy (fNIRS) study. IEEE Trans Games.

[7] Rahman MA, Siddik AB, Ghosh TK, Khanam F, Ahmad M (2020). A narrative review on clinical applications of fNIRS. J Digit Imaging.

[8] Wolf M, Naulaers G, Van Bel F, Kleiser S, Greisen G (2012). A review of near infrared spectroscopy for term and preterm newborns. J Near Infrared Spectrosc.

[9] Scholkmann F, Wolf M (2012). Measuring brain activity using functional near infrared spectroscopy: a short review. Spectrosc Eur [Internet]. https://www.spectroscopyeurope.com/system/files/pdf/NIR-24_4.pdf.

[10] Toronov V, Walker S, Gupta R, Choi JH, Gratton E, Hueber D, Webb A (2003). The roles of changes in deoxyhemoglobin concentration and regional cerebral blood volume in the fMRI BOLD signal. Neuroimage.

