# Supplementary material for: Feasibility of utilizing functional near-infrared spectroscopy to measure the cognitive load of paramedicine students undertaking high-acuity clinical simulations in Australia: a case study
Source: J Educ Eval Health Prof. 2024 Dec 10;21:38. doi: 10.3352/jeehp.2024.21.38 (PMC11717433; doi:10.3352/jeehp.2024.21.38)
Supplement: Supplementary file 2 — Supplement 1. Standardized objective structured clinical exam (OSCE) grading template. [file jeehp-21-38-suppl1.docx]

**Supplement 1.** Standardized objective structured clinical exam grading template.

**Exclusion criteria**

To provide a consistent level of academic ability and required clinical knowledge, only final year students with a grade point average (GPA) above 5.0 were considered for inclusion. Published exclusion criteria for participants included having been diagnosed anxiety or stress-related disorders, and taking medication which affects the central nervous system or cardiovascular system that could alter normal physiological responses to stress (e.g., medications for epilepsy, anxiety, mood disorders, and sleeping tablets such as Stilnox, benzodiazepines, melatonin, or beta blockers). Applying these criteria excluded 3 potential volunteers. Participants registered their interest via an online anonymized research participation form and were contacted by phone to discuss the research project and their eligibility for the study assessed.

**Chronic stress assessment**

To assess for chronic stress and burnout, participants completed a modified Copenhagen Burnout Inventory (CBI) before scenario 1. The 20-point CBI is a widely used tool for assessing burnout across various professions, including paramedicine [S1]. The validated CBI comprises 3 scales which measure personal burnout, work-related burnout, and education-related burnout [S2]. Each scale is scored as a percentage of time the participant reports feeling tired or exhausted in a variety of settings. Scores are tallied with an overall score out of 100. There is no consensus on CBI scores and level of burnout; however, literature suggests that scores below 40 are indicative of low burnout, 40–50 moderate burnout, and scores above 50 are considered to demonstrate a high burnout score [S3,S4].

**Acute stress assessment**

The Stressor Appraisal Scale (SAS), validated and described by Schneider [S5], is a tool used to determine acute cognitive stress relevant to an upcoming task. The SAS, completed prior to each scenario, consists of 7 primary (demand) and 3 secondary (resources) appraisal items recorded on a 7-point Likert scale. Recommendations from Hase et al. [S6] suggest calculating a differential between the demand and resource scores as a means to determine acute stress. Vine et al. [S7] support this noting that direct ratio scores often produce highly nonlinear distribution. For data presented within our study where the differential value (demand score minus resource score) was higher than the resources score, an elevated acute stress level was determined. To ensure responses obtained from the questionnaire reflected acute stress and not anticipation stress, the SAS was repeated immediately after each scenario with results correlated.

**fNIRS instrumentation and data processing**

Numerous studies have demonstrated the effectiveness of functional near-infrared spectroscopy (fNIRS) in various applications, including cognitive neuroscience [S8], clinical research [S9,S10], and brain-computer interface studies [S11]. Al-Shargie et al. [S12] and Rosenbaum et al. [S13] demonstrated that fNIRS was able to quantify mental stress, whilst Shirvan et al. [S14] concluded that signals recorded from only one site in the prefrontal cortex were able to effectively classify stress levels. fNIRS is considered a reliable method for neural monitoring in certain contexts, with advantages and limitations that should be considered depending on the type of research, clinical application, or specific brain regions under investigation. From a healthcare education perspective, complex cognitive tasks within simulated healthcare learning environments are known to increase cognitive load and mental effort [S15]. The fNIRS modality may be suited to assessing cognitive load of participants when performing prolonged real-time adaptations and complex psychomotor skills in these simulations [S16,S17].

The 6-channel continuous wave PortaLite MKII (Artinis Medical Systems) fNIRS system utilizes the modified Beer-Lambert law with an age-dependent differential pathlength to map O_2_Hb, HHb, and total Hb concentration changes within cortical brain tissue. Sensors consisting of 3 light-emitting diodes, each with 2 wavelengths of 760 and 850 nm, and 2 photodiode receivers were placed approximately 2 cm above the right eyebrow as recommended by Artinis Medical Systems.

This location assesses blood flow to the prefrontal cortex via inter-optode distance of 29, 35, and 41 mm with a sample rate 100 Hz. Three channels each of O_2_Hb and HHb were acquired and processed via Oxysoft software ver. 3.2.72 (Artinis Medical Systems). A black sports headband covered the sensors to prevent ambient light interference, and the control unit and battery were placed in a belt around the waist. To obtain baseline, all participants were asked to sit perfectly still with their head straight and upright for 30 seconds prior to scenario commencement. Mean values were determined for the 3 channels of O_2_Hb and HHb and all data reported as variation from baseline.

Room atmospheric conditions were controlled to be as similar as possible. Temperature (°C), relative humidity (%), ambient noise (dB), and illumination (lux) were all recorded and monitored via a handheld 5-in-1 meter (model: LM-8102; Munro Instruments).

**Statistical analysis**

Statistical analyses were conducted using IBM SPSS ver. 29.0 (IBM Corp.; running on Windows 10). All data were reported as means±standard deviation and P<0.05 was deemed statistically significant. Paired Samples t-tests with bootstrapping were undertaken for statistical analysis of data within a scenario, and these tests were applied to early, mid, and late time-points within the time series of data for each key variable.

**Limitations**

Our small sample size of n=8 restricted any definitive statistically significant findings. However, with each participant completing 2 separate scenarios and the PortaLite MKII recording 6 channels (3 O_2_Hb and 3 HHb), we were able to gather 96 individual wavelengths of neural activity data. Each wavelength recorded a data point every 1/10th of second over an average scenario time of 30.8 minutes, meaning an exceptionally large pool of data was available for analysis.

As a relatively new technology, some key limitations must be also considered. The spatial resolution of fNIRS is not as high as some other neuroimaging techniques like functional magnetic resonance imaging. This means that it may not provide precise information about the location of neural activity. Also, near-infrared light used in fNIRS has limited penetration depth, mainly capturing signals from the cortical surface. This restricts the ability to study deeper brain structures. The signals measured by fNIRS are also influenced by scalp and skull tissue with variability in individual anatomy potentially affecting the accuracy of measurements.

The learning environment may also be a confounder to stress levels. If any participant had a previous objective structured clinical exam fail result in the same laboratory space for which this experiment was undertaken, a priori experience may cause elevated stress responses due to environmental triggers which could potentially affect results.

**References**

S1. Crowe RP, Bower JK, Cash RE, Panchal AR, Rodriguez SA, Olivo-Marston SE. Association of burnout with workforce-reducing factors among EMS professionals. Prehosp Emerg Care 2018;22:229-236. <https://doi.org/10.1080/10903127.2017.1356411>

S2. Thrush CR, Gathright MM, Atkinson T, Messias EL, Guise JB. Psychometric properties of the Copenhagen Burnout Inventory in an academic healthcare institution sample in the U.S. Eval Health Prof 2021;44:400-405. <https://doi.org/10.1177/0163278720934165>

S3. Caesar B, Barakat A, Bernard C, Butler D. Evaluation of physician burnout at a major trauma centre using the Copenhagen Burnout Inventory: cross-sectional observational study. Ir J Med Sci. 2020 Nov;189(4):1451-1456. <https://doi.org/10.1007/s11845-020-02223-5>

S4. Reardon M, Abrahams R, Thyer L, Simpson P. Review article: Prevalence of burnout in paramedics: a systematic review of prevalence studies. Emerg Med Australas 2020;32:182-189. <https://doi.org/10.1111/1742-6723.13478>

S5. Schneider TR. Evaluations of stressful transactions: what’s in an appraisal? Stress Health 2008;24:151-158. <https://doi.org/10.1002/smi.1176>

S6. Hase A, O’Brien J, Moore LJ, Freeman P. The relationship between challenge and threat states and performance: a systematic review. Sport Exerc Perform Psychol 2019;8:123-144. <https://doi.org/10.1037/spy0000132>

S7. Vine SJ, Freeman P, Moore LJ, Chandra-Ramanan R, Wilson MR. Evaluating stress as a challenge is associated with superior attentional control and motor skill performance: testing the predictions of the biopsychosocial model of challenge and threat. J Exp Psychol Appl 2013;19:185-194. <https://doi.org/10.1037/a0034106>

S8. Carrieri M, Petracca A, Lancia S, Basso Moro S, Brigadoi S, Spezialetti M, Ferrari M, Placidi G, Quaresima V. Prefrontal cortex activation upon a demanding virtual hand-controlled task: a new frontier for neuroergonomics. Front Hum Neurosci 2016;10:53. <https://doi.org/10.3389/fnhum.2016.00053>

S9. Xu G, Huo C, Yin J, Zhong Y, Sun G, Fan Y, Wang D, Li Z. Test-retest reliability of fNIRS in resting-state cortical activity and brain network assessment in stroke patients. Biomed Opt Express 2023;14:4217-4236. <https://doi.org/10.1364/BOE.491610>

S10. Ranchet M, Hoang I, Derollepot R, Paire-Ficout L. Between-sessions test-retest reliability of prefrontal cortical activity during usual walking in patients with Parkinson’s disease: a fNIRS study. Gait Posture 2023;103:99-105. <https://doi.org/10.1016/j.gaitpost.2023.05.003>

S11. Naseer N, Hong KS. fNIRS-based brain-computer interfaces: a review. Front Hum Neurosci 2015;9:3. <https://doi.org/10.3389/fnhum.2015.00003>

S12. Al-Shargie F, Tang TB, Kiguchi M. Stress assessment based on decision fusion of EEG and fNIRS signals. IEEE Access 2017;5:19889-19896. <https://doi.org/10.1109/ACCESS.2017.2754325>

S13. Rosenbaum D, Hilsendegen P, Thomas M, Haeussinger FB, Metzger FG, Nuerk HC, Fallgatter AJ, Nieratschker V, Ehlis AC. Cortical hemodynamic changes during the Trier Social Stress Test: an fNIRS study. Neuroimage 2018;171:107-115. <https://doi.org/10.1016/j.neuroimage.2017.12.061>

S14. Shirvan RA, Setaredan SK, Nasrabadi AM. Classification of mental stress levels by analyzing fNIRS signal using linear and non-linear features. Int Clin Neurosci J 2018;5:55-61. <https://doi.org/10.15171/icnj.2018.11>

S15. Taylor N, Wyres M, Bollard M, Kneafsey R. Use of functional near-infrared spectroscopy to evaluate cognitive change when using healthcare simulation tools. BMJ Simul Technol Enhanc Learn 2020;6:360-364. <https://doi.org/10.1136/bmjstel-2019-000517>

S16. Sevcenko N, Schopp B, Dresler T, Ehlis AC, Ninaus M, Moeller K, Gerjets P. Neural correlates of cognitive load while playing an emergency simulation game: a functional near-infrared spectroscopy (fNIRS) study. IEEE Trans Games 2022;14:696-705. <https://doi.org/10.1109/TG.2022.3142954>

S17. Toy S, Huh DD, Materi J, Nanavati J, Schwengel DA. Use of neuroimaging to measure neurocognitive engagement in health professions education: a scoping review. Med Educ Online 2022;27:2016357. <https://doi.org/10.1080/10872981.2021.2016357>
